# Synaptic Function and Sensory Processing in *ZDHHC9*‐Associated Neurodevelopmental Disorder: A Mechanistic Account

**DOI:** 10.1111/ejn.70124

**Published:** 2025-05-01

**Authors:** Rebeca Ianov Vitanov, Jascha Achterberg, Danyal Akarca, Duncan E. Astle, Kate Baker

**Affiliations:** ^1^ MRC Cognition and Brain Sciences Unit University of Cambridge Cambridge UK; ^2^ Department of Psychiatry University of Cambridge Cambridge UK; ^3^ Department of Medical Genetics University of Cambridge Cambridge UK

**Keywords:** ZDHHC9, intellectual disability, epilepsy, language, MEG, recurrent neural networks

## Abstract

Loss‐of‐function *ZDHHC9* variants are associated with X‐linked intellectual disability (XLID), rolandic epilepsy (RE) and developmental language difficulties. This study integrates human neurophysiological data with a computational model to identify a potential neural mechanism explaining *ZDHHC9*‐associated differences in cortical function and cognition. Magnetoencephalography (MEG) data was collected during an auditory roving oddball paradigm from eight individuals with a *ZDHHC9* loss‐of‐function variant (ZDHHC9 group) and seven age‐matched individuals without neurological or neurodevelopmental difficulties (control group). Auditory‐evoked fields (AEFs) were larger in amplitude and showed a later peak latency in the ZDHHC9 group but demonstrated normal stimulus‐specific properties. Magnetic mismatch negativity (mMMN) amplitude was also increased in the ZDHHC9 group, reflected by stronger neural activation during deviant processing relative to the standard. A recurrent neural network (RNN) model was trained to mimic group‐level auditory‐evoked responses, and subsequently perturbed to test the hypothesised impact of *ZDHHC9*‐driven synaptic dysfunction on neural dynamics. Results of model perturbations showed that reducing inhibition levels by weakening inhibitory weights recapitulates the observed group differences in evoked responses. Stronger reductions in inhibition levels resulted in increased peak amplitude and peak latency of RNN prediction relative to the pre‐perturbation predictions. Control experiments in which excitatory connections were strengthened by the same levels did not result in consistently stable activity or AEF‐like RNN predictions. Together, these results suggest that reduced inhibition is a plausible mechanism by which loss of ZDHHC9 function alters cortical dynamics during sensory processing.

## Graphical abstract

In the current study, we employed a bottom‐up approach to study the impact of synaptic‐level alterations associated with *ZDHHC9* variants on cortical function in healthy and *ZDHHC9*‐deficient participants. To achieve this, a recurrent neural network model was employed to recapitulate MEG‐derived auditory‐evoked responses and subsequently perturbed in order to determine effects on resulting dynamics. We showed that reduced network inhibition recapitulates trends observed empirically, specifically increased response amplitudes, delayed peak latencies and increased mismatch negativity. These results offered a mechanistic account on the impact of *ZDHHC9*‐associated synaptic alterations on auditory processing.

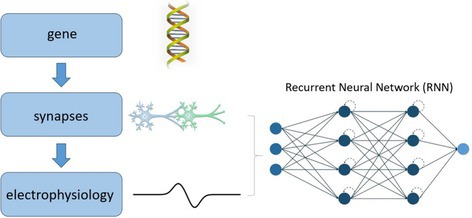

**Abbreviations**
Adam: Adaptive moment estimation
AEF: Auditory‐evoked field
D: Deviant (stimulus)
E:I: Excitatory‐to‐inhibitory
fT: Femtotesla
FWER: Family‐wise error rate
GABA: Gamma‐aminobutyric acid
HEOG: Horizontal electrooculogram
Hz: Hertz
HPI: Head position indicator
ICA: Independent component analysis
ID: Intellectual disability
IQ: Intelligence quotient
MAE: Mean absolute error
MEG: Magnetoencephalography
MMN: Mismatch negativity
mMMN: Magnetic mismatch negativity
MRI: Magnetic resonance imaging
MNE: Magnetoencephalography and EEG analysis toolbox
MSE: Mean squared error
PCA: Principal component analysis
PSD‐95: Post‐synaptic density protein 95
R1: First repeat
RE: Rolandic epilepsy
ReLU: Rectified linear unit
RNN: Recurrent neural network
s.d.: Standard deviation
S: Standard (stimulus)
STFT: Short‐time Fourier transform
VEOG: Vertical electrooculogram
WASI‐II: Wechsler Abbreviated Scale of Intelligence—Second Edition
XLID: X‐linked intellectual disability
ZDHHC9: Zinc finger DHHC‐type palmitoyltransferase 9

## 1 Introduction

Cognition is sculpted during development by a myriad of genetic, cellular and systems‐level mechanisms. Studying rare single gene disorders related to intellectual disability (ID), in combination with neural network models of cognition, could provide insights into specific mechanisms contributing to developmental cognitive difficulties. When made computationally tractable, specific cellular and systems‐level mechanisms associated with genes of interest might offer explanatory support for the aetiology of cognitive impairment. Additionally, simplified computational models of the brain can be trained to perform tasks and then systematically perturbed to recreate ‘disorder‐like’ model predictions. This framework is in line with the theoretical view that genes, brains, and artificial neural networks have similar underlying goals, which are to maximise the probabilities of achieving objectives, whether these be protein function, neural systems and cognitive functions, or good task performance, respectively (Kamatani 2021). However, this approach has not previously been applied to rare genetic disorders, owing to the limited data availability across levels for these groups, particularly functional neuroimaging at appropriate temporal resolution. In this study, we trial the approach by employing a neural network model of auditory processing as a tool for mapping genetically driven, local alterations to systems‐level activity, in a group of individuals with cognitive impairment of known genetic origin.

A relevant gene for studying the emergence of intellectual disabilities is *ZDHHC9*, which encodes a palmitoylation enzyme (ZDHHC9) involved in the post‐translational modification and intracellular trafficking of specific target substrates (Topinka and Bredt 1998; Fukata and Fukata 2010). Loss of function *ZDHHC9* variants have been associated with mild to moderate ID (Fukata and Fukata 2010), oromotor speech difficulties and language impairments (Baker et al. 2015), often coexisting with rolandic seizures (Baker et al. 2015; Shimell et al. 2019). Comorbidity between rolandic seizures and language difficulties has been commonly observed in non‐ZDHHC9 cohorts, but the mechanisms linking these symptoms remain elusive (Smith et al. 2012; Clarke et al. 2007). Hence, discovery of a rare monogenic cause of these associations may highlight specific, symptom‐relevant neurobiological processes.

MRI studies of individuals with *ZDHHC9*‐associated ID identified neuroanatomical differences that may increase the risk for epilepsy and cognitive impairments, such as reductions in cortical thickness and connectomic deviations (Baker et al. 2015; Bathelt et al. 2016; Bathelt et al. 2017). Another study of the same participant group employed resting‐state magnetoencephalography (MEG) and revealed differences in state activation duration as well as dynamic connectivity across networks, with the extent of case–control differences correlating with *ZDHHC9* expression levels (Hawkins et al. 2020). While the studies outlined above have described the neurological, behavioural, neuroanatomical and global MEG characteristics of *ZDHHC9*‐associated XLID, local MEG characteristics and causal links remain unexplored.

At the molecular and cellular level, experimental studies of *ZDHHC9* loss‐of‐function point toward developmental differences in synaptic structure and function. Targets of the ZDHHC9 enzyme include the GTPase Ras, which promotes dendrite outgrowth, as well as GTPase TC10, which supports inhibitory synapse formation (Shimell et al. 2019). A study of the impact of *ZDHHC9* loss‐of‐function in primary rat hippocampal cultures revealed shorter and less complex dendritic arbours and an increase in the ratio of excitatory‐to‐inhibitory (E:I) synapses (Shimell et al. 2019). Moreover, *ZDHHC9* knockout mice showed spontaneous high‐frequency spiking activity potentially reflecting non‐convulsive seizures (Shimell et al. 2019). Another important target of ZDHHC9 is post‐synaptic density protein 95 (PSD‐95), a synaptic scaffolding protein that plays a key role in bidirectional synaptic plasticity, which is essential for learning and memory (Wu et al. 2017). In summary, there is emerging evidence that *ZDHHC9* variants alter properties of neuronal development and plasticity important for maintaining synaptic E:I balance and, ultimately, optimal neural function.

The current study aimed to link *ZDHHC9*‐associated ID participants' neurophysiology to previously reported cellular and synaptic differences, within a computational framework. MEG data were recorded from participants with *ZDHHC9* variants and control participants during a passive roving oddball paradigm. This enabled assessment of auditory change detection via MEG mismatch negativity (mMMN), an auditory‐evoked field reflecting brain response differences to stimuli with distinct properties without requiring directed attention (Cowan et al. 1993; Garrido et al. 2009). A roving protocol was designed for the study since this is an efficient method for observing mismatch responses and is relatively independent of basic stimulus properties. In addition, the roving oddball paradigm has been widely used in MEG studies of clinical groups with cognitive impairment (Kirihara et al. 2020; Näätänen et al. 2014).

Taking inspiration from previous studies integrating neural network modelling with electrophysiological data (O'Reilly 2022; O'Reilly, Angsuwatanakul, and Wehrman 2022), we employed a recurrent neural network to test a causal model relating *ZDHHC9*‐related synaptic alterations to observed differences in group‐level auditory‐evoked fields (AEFs).

## 2 Materials and Methods

### 2.1 Participants

Eight male participants age 9–41 with ZDHHC9‐associated X‐linked ID were recruited to the study (ZDHHC9 group), having received their genetic diagnosis within the GOLD (Genetics of Learning Disability) study which included permission for research re‐contact (Raymond et al. 2007). Recruitment process and assessment methods plus clinical and cognitive characteristics of this group have been previously described (Baker et al. 2015). Mean estimated full scale IQ was 65 (standard deviation 6), assessed via the four sub‐test WASI‐II (Carney 2011). Seven individually age‐matched male comparison participants were recruited, free of neurological and psychiatric disorders (control group). Informed consent was obtained from each participant or their parent/consultee. Ethical approval for the study was granted by the Cambridge Central Research Ethics Committee (11/0330/EE).

### 2.2 Data Acquisition

All MEG datasets were collected on a 306‐channel high‐density whole‐head VectorView MEG system (Elekta Neuromag, Helsinki), consisting of 102 magnetometers and 204 orthogonal planar gradiometers, located in a light magnetically shielded room. Data were sampled at 1 kHz and signals slower than 0.01 Hz were not recorded (online filter applied). A 3D digitizer (FASTRACK; Polhemus) was used to record the positions of five head position indicator (HPI) coils and 50–100 additional points evenly distributed over the scalp, all relative to the nasion and left and right preauricular points. An electrode was attached to each wrist to measure the pulse and bipolar electrodes to obtain horizontal (HEOGs) and vertical (VEOGs) electrooculograms. Head position was monitored throughout the recording using the HPI coils.

### 2.3 Stimuli and Design

**FIGURE 1:**
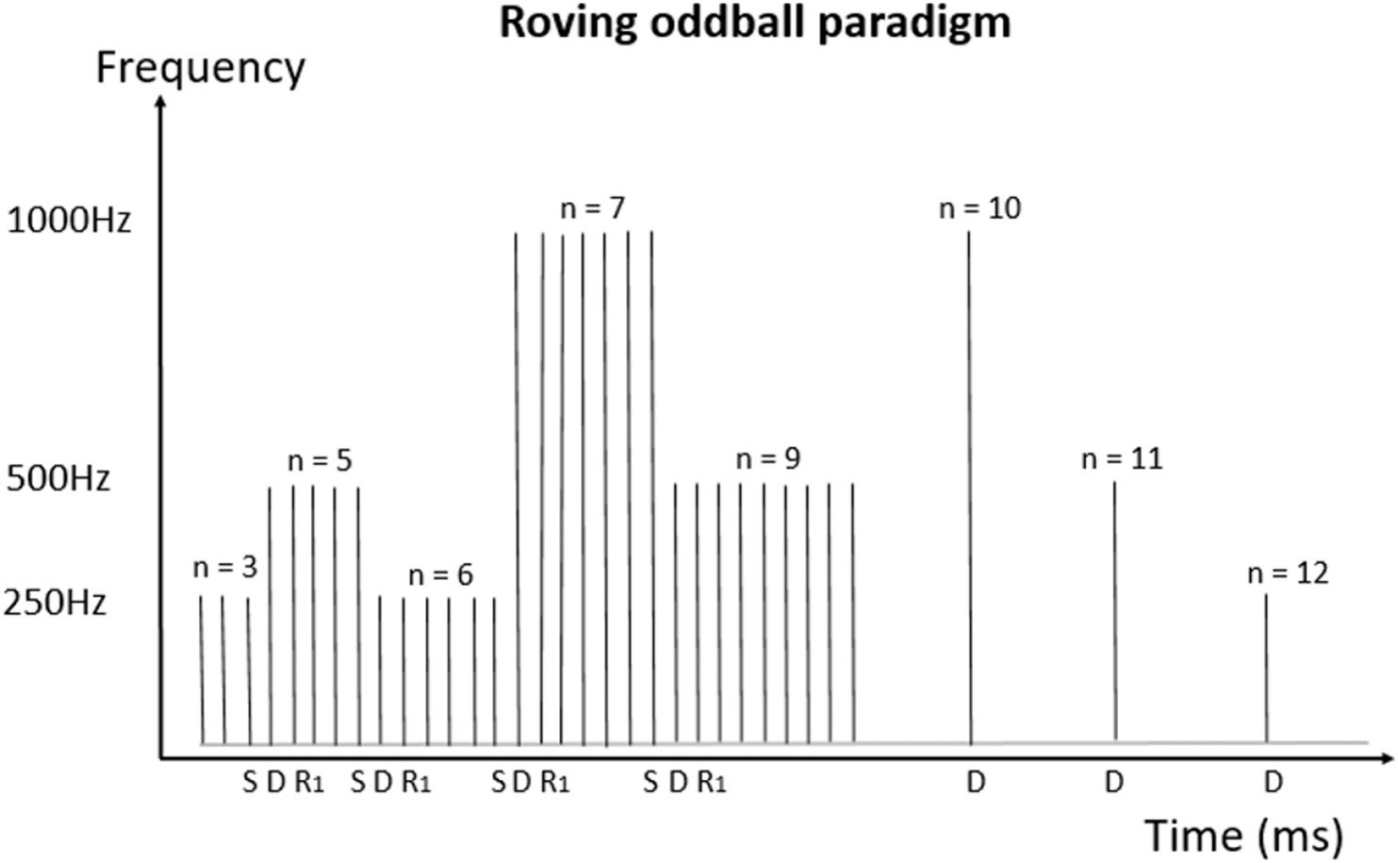
Roving oddball paradigm. A standard tone (S) of either 250 Hz, 500 Hz, or 1000 Hz is presented randomly between 3 and12 times (n). After this sequence of repetitions, the frequency of the tone changes (deviant tone, D), which then becomes the new standard through repetitions (R_1_ denotes the first repeat). In the right part of the figure, the first tones (deviants) of new series of tones (10, 11, or 12 presentations) are depicted (subsequent repeats are not drawn for simplicity).

The experimental design was based on a roving oddball paradigm described by Cowan et. al (Cowan et al. 1993). This experimental scheme involved the repeated presentation of between 3 and 12 identical ‘standard’ stimuli of a particular frequency (250 Hz, 500 Hz or 1000 Hz) followed by a ‘deviant’ tone of a different frequency to the previous standard stimuli. In turn, the deviant is repeated and becomes the new standard (Figure 1). Tones were 50 ms in duration and the inter‐tone interval was fixed at 500 ms (550 ms stimulus onset asynchrony). Participants were instructed to watch a silent movie and ignore the tones.

### 2.4 MEG Data pre‐Processing

The raw MEG data was pre‐processed with the MNE package version 1.0.3 (Gramfort et al. 2013) in Python 3.10 (Rossum and Drake 2009). External noise was removed using a signal‐space separation method and adjustments in head position within the recording were compensated for using Maxwell filtering. A sensor‐space temporal independent components analysis (ICA) was used to automatically remove artefacts arising from blinks, saccades and pulse‐related cardiac artefacts, and the outputs were manually checked by visual inspection. Data were epoched to a time window defined as 400 ms pre‐stimulus and 550 ms post‐stimulus to ensure all relevant event‐related changes were contained within the epoch time window, and then down‐sampled to 250 Hz, baseline corrected and low‐pass filtered at 30 Hz utilising a 4th order Butterworth filter. All trials with larger peak‐to‐peak amplitude than 4e‐12 Tesla (4000 fT) and smaller peak‐to‐peak amplitude than 1fT at each magnetometer were removed. For each participant, data were averaged across all trials to form the time‐domain signals. AEF analyses were also performed with the MNE package (version 1.0.3; (Gramfort et al. 2013)).

### 2.5 Data Analysis

AEFs across all stimuli types were computed for both groups. The grand average of all trial types was used to compute AEF time‐domain signals. Peak amplitude and peak latencies of AEFs were automatically calculated at the magnetometer where the largest amplitude signal was detected. The precise latencies of M100 responses are useful indicators of the temporality of auditory processing (Gage, Siegel, and Roberts 2003). To account for a consistent 50 ms delay in the tone stimulus presentation relative to the trigger timing in the scanner setup, a peak of activity at approximately 150 ms post‐trigger was automatically detected as equivalent to the widely reported M100 response (Roberts et al. 2000).

Differences in activity elicited by standard and deviant stimuli were analysed for both groups using non‐parametric cluster‐based permutation testing (Maris and Oostenveld 2007). This method addresses the issue of numerous multiple comparisons by controlling the family‐wise error rate (FWER) at the level of clusters of adjacent data points (spatially and temporally) rather than individual data points (Maris and Oostenveld 2007). To generate a null distribution under the null hypothesis, condition labels and time points were permuted across trials. For each permutation, clusters of adjacent data points exceeding a predefined threshold (cluster forming test statistic threshold = 3.3) were identified. The observed cluster‐level statistics from the original (non‐permuted) data were then compared against this null distribution, using a significance threshold of α = 0.05. The entire epochs from all trials were used as input to the algorithm. The cluster‐based permutation test identified the sensor locations and timeframes for each condition where significant differences arose. Deviant responses (D) were compared to their preceding standard stimuli (S), and the mMMN was calculated as the mean absolute error (MAE) between standard‐ and deviant‐evoked responses at the group level. Individual MMNs were calculated at the largest cluster significant at the group level.

### 2.6 Neural Network Modelling

A recurrent neural network (RNN) consisting of an input layer (63 units), four hidden layers (each with 64 recurrent units) and an output layer (1 recurrent unit) was employed as a model of auditory processing. RNNs are a class of artificial neural networks where the connections between nodes can create a cycle, allowing output from some nodes to be fed as input to the same nodes. These recurrent connections allow RNNs to learn from sequences of inputs and are loosely analogous to feedback connections in biological neural networks (Rumelhart, Hinton, and Williams 1988). Each RNN node is analogous to a population of neurons, which can emit excitatory or inhibitory connections or weights (Guresen and Kayakutlu 2011). The model used is a discrete‐time RNN, which processed the input at each timestep according to the recurrence formula (Equation 1), where the matrix $W_{\mathrm{hh}}$ captures the recurrent connections, $h_{t-1}$ is the previous hidden states vector, $W_{\mathrm{xh}}$ is the feedforward weight matrix, the $x_{t}$ is the input vector at timestep t and $b_{h}$ is the bias vector. The RNN output at each timestep is obtained according to Equation 2, where $W_{\mathrm{hy}}$ is the weight matrix between the last hidden layer and the output units, $h_{t}$ is the hidden state vector at the current timestep and $b_{y}$ is the bias vector (Mienye, Swart, and Obaido 2024).

(1) $$h\left(t\right)=\text{ReLU}\left(W_{\mathrm{hh}}h_{t-1}+W_{\mathrm{xh}}x_{t}+b_{h}\right)$$

(2) $$y\left(t\right)=W_{\mathrm{hy}}h_{t}+b_{y}$$

The RNN was trained in a supervised fashion, and the labels (targets) were obtained from the control group cluster (12 channels in the right hemisphere) where significant S‐D differences were detected. The average across these channels was computed to obtain a group‐level AEF for each stimulus type (S and D). Gaussian white noise (mean = 0, s.d. = 0.6) was then added to each of the two AEFs and 1200 labels were generated for each stimulus condition. Before feeding the labels into the RNN model, the data was robust‐scaled and flipped so that most values are above 0. Robust scaling sets the median and interquartile range to 0 and 1, respectively, and therefore maintains the directionality of the amplitude difference between S and D trial type AEFs (i.e. the RNN predictions when D inputs are given have a higher amplitude than the S predictions, as observed empirically).

The RNN inputs were sound waveforms produced with a sampling frequency of 5000 Hz. Standard or deviant inputs to the RNN were defined in relation to the precedent tone frequency. A standard (S) input to the RNN consisted of a spectrogram with 3 sinewaves of the same frequency, whereas a deviant (D) input consisted of a spectrogram with the first 2 sinewaves of the same frequency and the third one of a different frequency. The RNN model thus learns from stimulus history what the subsequent AEF is. The frequencies used for the sinewaves were 250 Hz, 500 Hz and 1000 Hz (Figure 1). These were converted into the time‐frequency domain using the short‐time Fourier transform (STFT), producing a representation of cortical input from the ascending auditory pathway (Rahman et al. 2020). The STFT was performed on Hann‐windowed segments of 125 samples with an overlap of 105 samples. Input features were fed through the model and its parameters (weights and biases) were optimised to minimise mean‐squared‐error (MSE) loss between model outputs at the current timestep, $f_{t}$, and target evoked responses, or observed values at the current timestep, $y_{t}$ as shown in Equation 3.

(3) $$\mathrm{MSE}=\frac{1}{N}\sum_{t=1}^{N}\left(f_{t}-y_{t}\right)$$

Adaptive moment estimation (Adam) optimization was used with a learning rate of 0.0002 and a dropout regularisation of 0.15 was used in the hidden layers. Each hidden layer had a rectified linear unit (ReLU) activation, whereas the final layer had a linear activation function. Connection weights between layers were initialised from a Glorot uniform distribution and recurrent weights were initialised as an orthogonal matrix from a normal distribution (Glorot and Bengio 2010; Saxe, McClelland, and Ganguli 2013).

A 70%:15%:15% train/validation/test split was used for training, tuning the model parameters (validation) and testing the model performance. Subsequently, three perturbation experiments were performed by systematically reducing inhibition levels after network training. In experiment 1 (‘negative weight perturbation’), the negative recurrent weights of the four hidden layers were reduced, in terms of absolute values, by eight incremental levels: 0.5%, 1%, 1.5%, 2%, 2.5%, 3%, 3.5%, 4%. Two additional control experiments were performed, in which the outcomes were assessed for increasing excitation or concomitantly increasing excitation and reducing inhibition, for the same levels as in the first experiment. In experiment 2 (‘positive weight perturbation’), the positive recurrent weights were relatively increased by 0.5–4% (with the same increments as in experiment 1). In experiment 3 (‘random weight perturbation’), a random set of recurrent weights, including both positive and negative connections, were altered by the same levels (positive weights were increased and the absolute value of negative weights was decreased).

The stability of RNN predictions in the three experiments was validated using principal component analysis (PCA). The units' activations of the fourth hidden layer, represented by a matrix of size (64 [units], 138 [timepoints]), were transformed into principal component spaces of size (2 [components], 138 [timepoints]). This transformation preserves as much variance as possible from data in the original matrix while compressing them into fewer columns. The resulting neural latent space, obtained after PCA, is shown on a cartesian plane in Figure S4, which indicates the activity in the last hidden layer over the timecourse of the RNN prediction.

## 3 Results

### 3.1 Comparative Analysis of AEF and MMN Responses in the Control and ZDHHC9 Groups

AEFs across all stimuli types were computed for the ZDHHC9 and control groups (Supplementary Figure S1). In the following results, AEF latencies are reported relative to the scanner trigger onset (0 ms). Due to a 50‐ms delay between the trigger and the actual stimulus presentation, the reported latencies are offset by 50 ms. This timing discrepancy should be considered when interpreting the latency values reported in this study. Mean peak activity occurred at 144 ms post‐trigger in the control group, reaching a peak absolute distribution of 181fT, which corresponded to an expected M100 response. In the ZDHHC9 group, mean peak activity over this window occurred at 203 ms post‐trigger (59 ms later in latency than controls; *t*_*11.9*_ = 1.9; *p* = 0.08), reaching a maximal absolute magnetic distribution at 192fT (1.06X greater in magnitude than controls; *t*_*12.8*_ = 0.9, *p* = 0.3). These observations remain qualitative as significant differences were not detected. The topographical plots computed at the peak latency (Supplementary Figure S1, right panel), indicated a clear dipolar pattern in both the left and right hemispheres in both groups.

Trial responses were then separated by frequency into 250 Hz, 500 Hz and 1000 Hz stimulus responses for both the control and ZDHHC9 group, to explore whether there was a systematic relationship between the frequency of the stimulus and the latency of the AEF. Given the spatial tonotopic organisation of the auditory cortex (Humphries, Liebenthal, and Binder 2010), groups of neurons that respond to higher frequency stimuli are activated before those that respond to lower frequency stimuli. Thus, it was expected that higher frequency stimuli would result in shorter latency responses (Humphries, Liebenthal, and Binder 2010; Pantev et al. 1988; Roberts and Poeppel 1996). In control participants, 250 Hz, 500 Hz and 1000 Hz stimuli evoked an AEF with a peak at 152 ms, 148 ms and 136 ms—decreasing in latency respectively as expected (Supplementary Figure S2). In the ZDHHC9 group, 250 Hz tones had a latency of 192 ms (40 ms later than controls; *t*_12.3_ = 0.3, *p* = 0.7), 500 Hz tones had a latency of 164 ms (16 ms later than controls; *t*_12.9_ = 2.4, *p* = 0.03), whereas 1000 Hz tones had a latency of 200 ms (64 ms later than controls; *t*_12.5_ = 3.1, *p* = 0.01). The two peaks present for the 1000 Hz stimuli were due to wider inter‐subject variability for the ZDHHC9 group. For all frequency stimuli, the responses in this group reflected a prolonged activation (Supplementary Figure S2).

**FIGURE 2:**
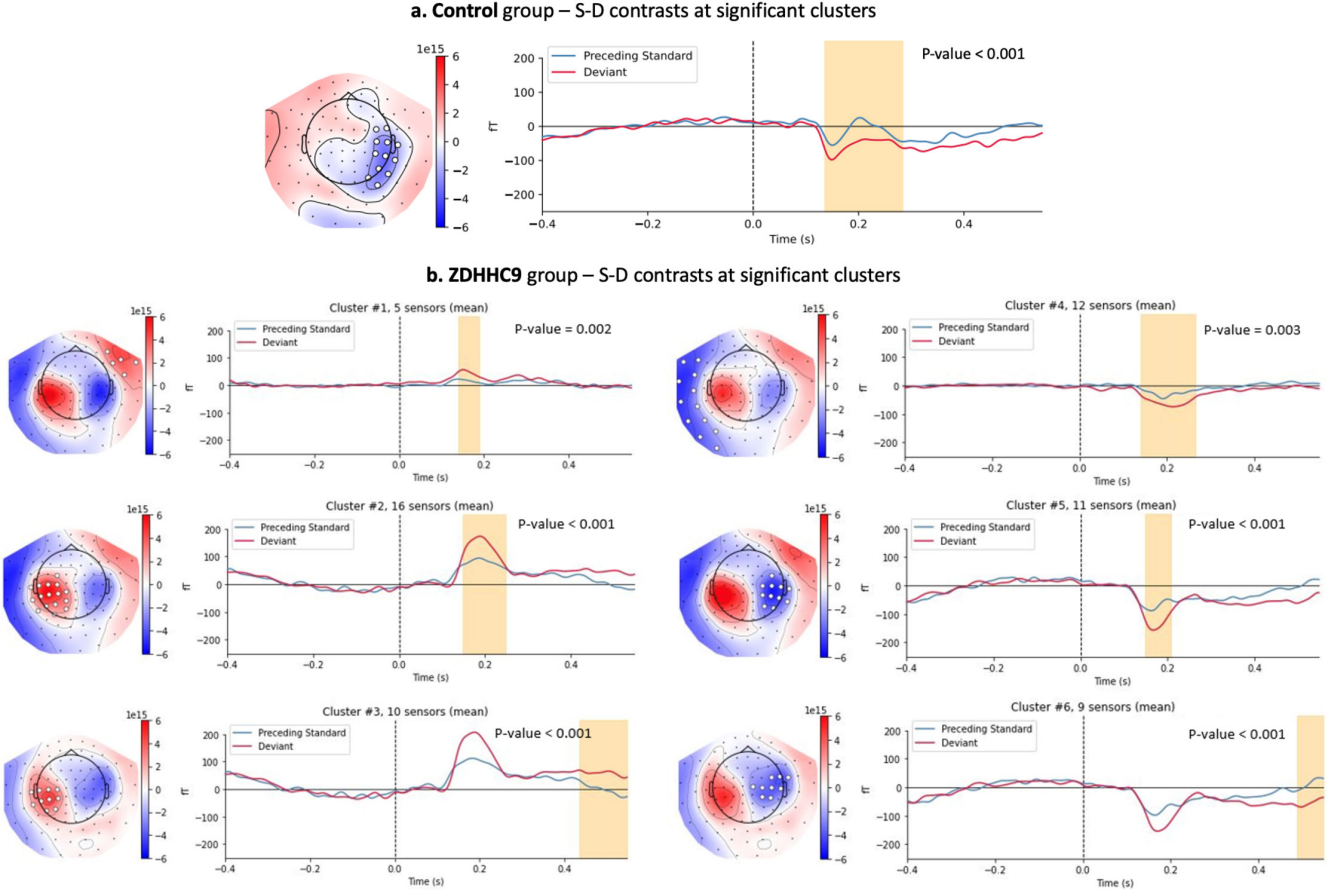
Cluster‐based permutation testing. Permutation‐based clusters of statistically significant differences between standard‐evoked and deviant‐evoked AEFs in control and ZDHHC9 groups. Time‐windows where these differences were identified are highlighted in yellow.

Next, responses to all deviants (D) were computed and compared to their preceding stimulus (S) to provide an index of the mMMN response across all standard train lengths, for the control and ZDHHC9 groups separately (Figure 2). Non‐parametric cluster‐based permutation testing revealed statistically significant differences between S and D trials (*p* = 0.0008) in the control group at a single negative cluster in the right hemisphere, occurring over 12 channels, from 150 to 180 ms after stimulus onset (Figure 2a). In the ZDHHC9 group, six significant clusters were identified, indicating that mismatch responses were topographically more extensive and of higher mean peak amplitude in the case group (Figure 2b). The responses of the two groups could only be directly compared by calculating the average evoked responses at the eight sensors where significant S‐D contrasts were found in both groups (Supplementary Figure S3). All averaged responses were significant within a time‐frame of an expected mMMN, around 100 ms after stimulus onset (corresponding to 150 ms post‐trigger). Individual‐level responses were computed for and averaged across these eight channels—differences in peak amplitudes between the two groups did not reach statistical significance for the standard‐evoked responses (*p* = 0.09) or the deviant‐evoked responses (*p* = 0.1).

### 3.2 Recurrent Neural Network Model of Neural Dynamics in Auditory Processing

**FIGURE 3:**
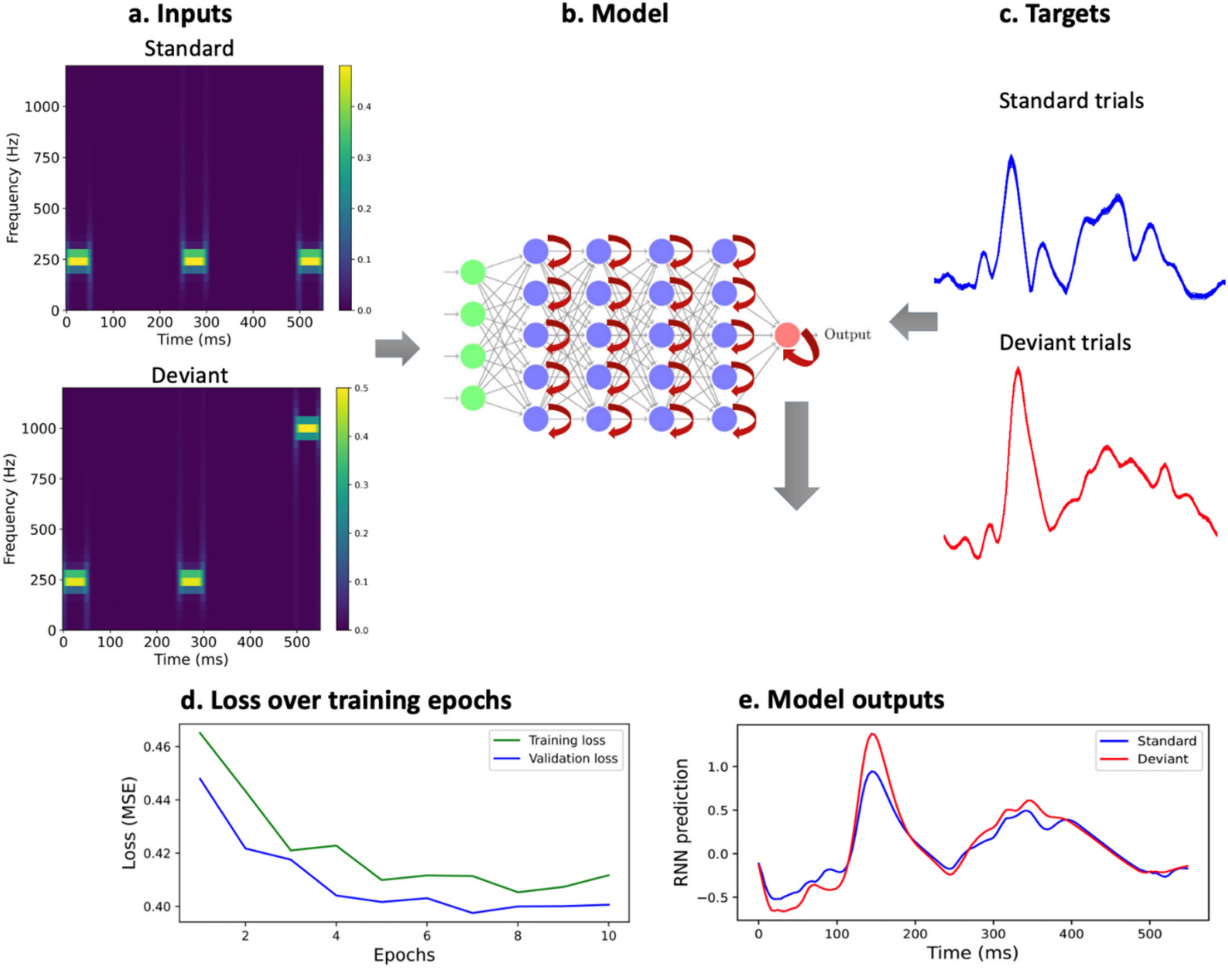
Overview of modelling workflow. (a) Spectrograms of example standard and deviant inputs used for the RNN. Three frequencies were used as in the roving oddball paradigm: 250 Hz, 500 Hz and 1000 Hz. A standard (S) input to the RNN model refers to a spectrogram with three identical tones. A deviant (D) input to the RNN model refers to a spectrogram with the third tone of a different frequency from the previous two. The train set consisted of S inputs of 250 Hz and 500 Hz, as well as the following sequences of tones, of which the third represented the D input: 250–250‐500 (Hz), 250–250‐1000 (Hz), 500–500‐1000 (Hz). The test set consisted of standard inputs of 1000 Hz and tone triads including: 500–500–250 (Hz), 1000–1000–250 (Hz), and 1000–1000–500 (Hz). Spectrograms spanned the same time range (548 ms) as the RNN labels (post‐trigger AEFs with a stimulus offset at 50 ms) and RNN outputs. Each tone in the spectrograms was 50 ms long, and inter‐tone intervals were 199 ms each. The RNN was trained to respond to the third tone in the sequence of three, depending on whether it is a standard or deviant. (b) Simplified diagram of the hierarchical RNN architecture. Input layer (green) had 63 recurrent units, each hidden layer had 64 units and the output layer had 1 recurrent unit. (c) Targets were 1200 simulated AEFs obtained by adding Gaussian white noise (standard deviation 0.6) to the control group level post‐stimulus AEF in response to standard tones. The same was done for deviant AEFs, resulting in 1200 simulated deviant AEFs. d. The RNN was trained for 10 epochs (i.e. iterations through the entire training dataset). The lower validation loss reflects the absence of dropout regularisation during validation, as opposed to training. e. RNN predictions to S and D inputs.

Figure 3 outlines the modelling workflow. Spectrograms consisting of three consecutive sinewaves were given to the RNN. The frequency of the last sinewave relative to the frequency of the previous two, which were always identical, determines whether the last tone is a standard or a deviant. A spectrogram represents a S input if all three tones have the same frequency and a D input if its third tone is of a distinct frequency than the previous two (Figure 3a). Empirically derived control group‐level AEFs, on which Gaussian noise was added, served as corresponding labels (Figure 3c). During training (10 epochs), MSE was minimised between the RNN predictions and the labels (Figure 3d). Average model outputs over all standard and deviant types, after training, are shown in Figure 3e.

**FIGURE 4:**
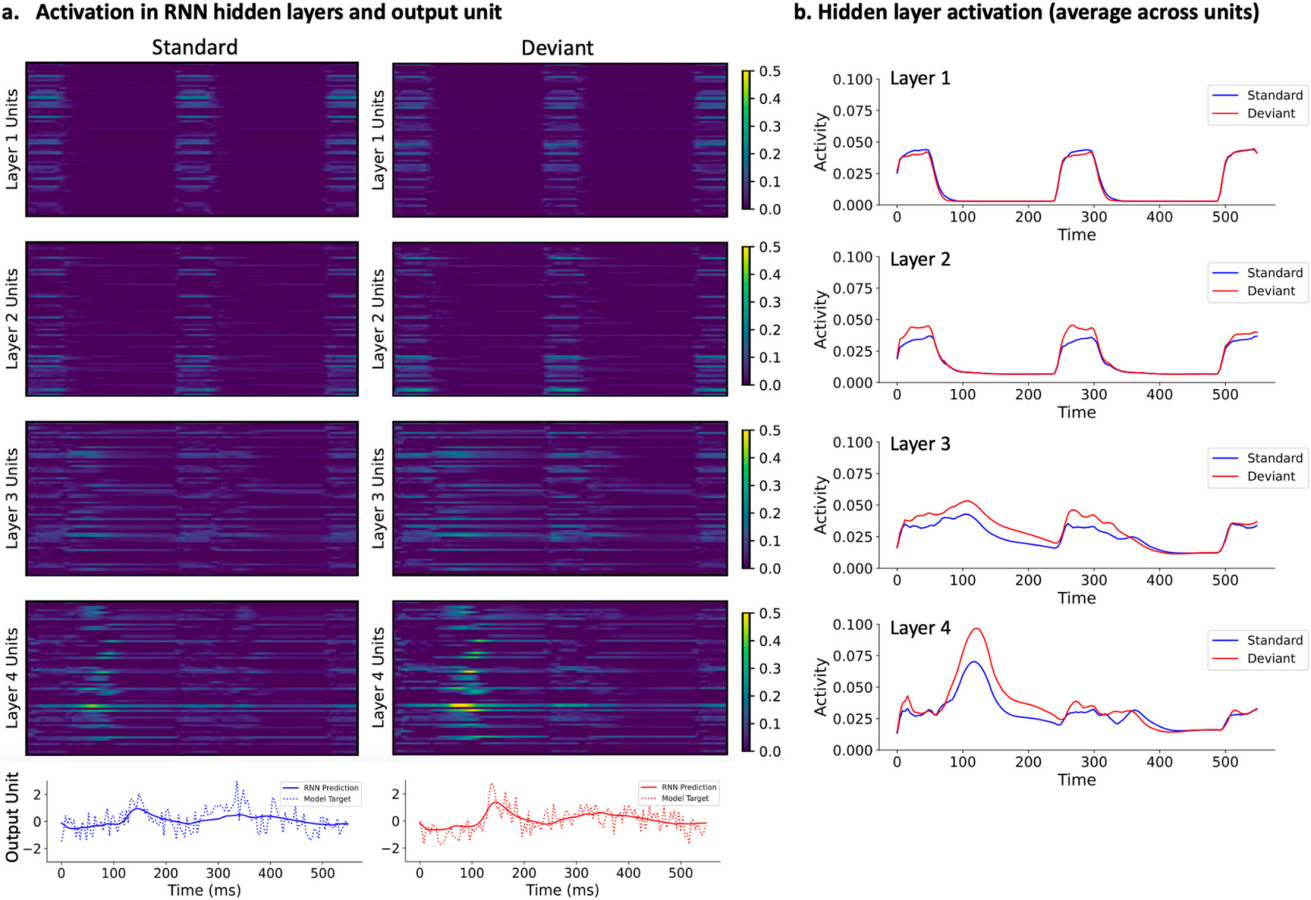
Hidden layer activations and output unit predictions for standard and deviant inputs. (a) Model predictions and hidden layer activations plotted by layer (rows) and input condition (columns). The activations were computed as an average across all S input types and all D input types, respectively. The *x*‐axis represents time. Amplitudes of hidden unit activations, shown in the four upper rows, increase toward the output. Patterns of hidden unit activations also spread out and become more complex with increasing layer depth. Visible differences in hidden unit response magnitudes between input conditions were also found. Model outputs and corresponding grand‐average AEFs (with Gaussian white noise; mean = 0 and s.d. = 0.6) are plotted in the bottom row. All data displayed in the panels represents the RNN response to the three‐tone input sequence (S or D). b. Hidden layer activations over time, averaged across the 64 hidden units.

To understand how the hierarchy of the RNN's layers corresponded to the types of inputs, we computed the RNN hidden layer activations for S and D inputs (Figure 4). This enabled us to qualitatively assess whether the network responded differentially based on the input and, if so, where in the network this was occurring. We found that network activity became gradually more diffuse in time from the first to the fourth hidden layers. In the first hidden layer, activations for the S and D inputs were largely similar, whereas in later layers of the hierarchy, the activity elicited by the D inputs became larger than that elicited by the S inputs (Figure 4a, b).

### 3.3 Reducing Inhibition Recapitulates Auditory Dynamics in the ZDHHC9 Group

To test the impact of alterations mimicking the *ZDHHC9* loss‐of‐function phenotype (i.e. reduced inhibition) on the RNN output, we conducted a perturbation experiment by systematically reducing inhibition levels after network training and observed the effects on the network's predictions. These perturbations altered the average excitation‐inhibition synapse ratio of ≈1:1 obtained after training.

**FIGURE 5:**
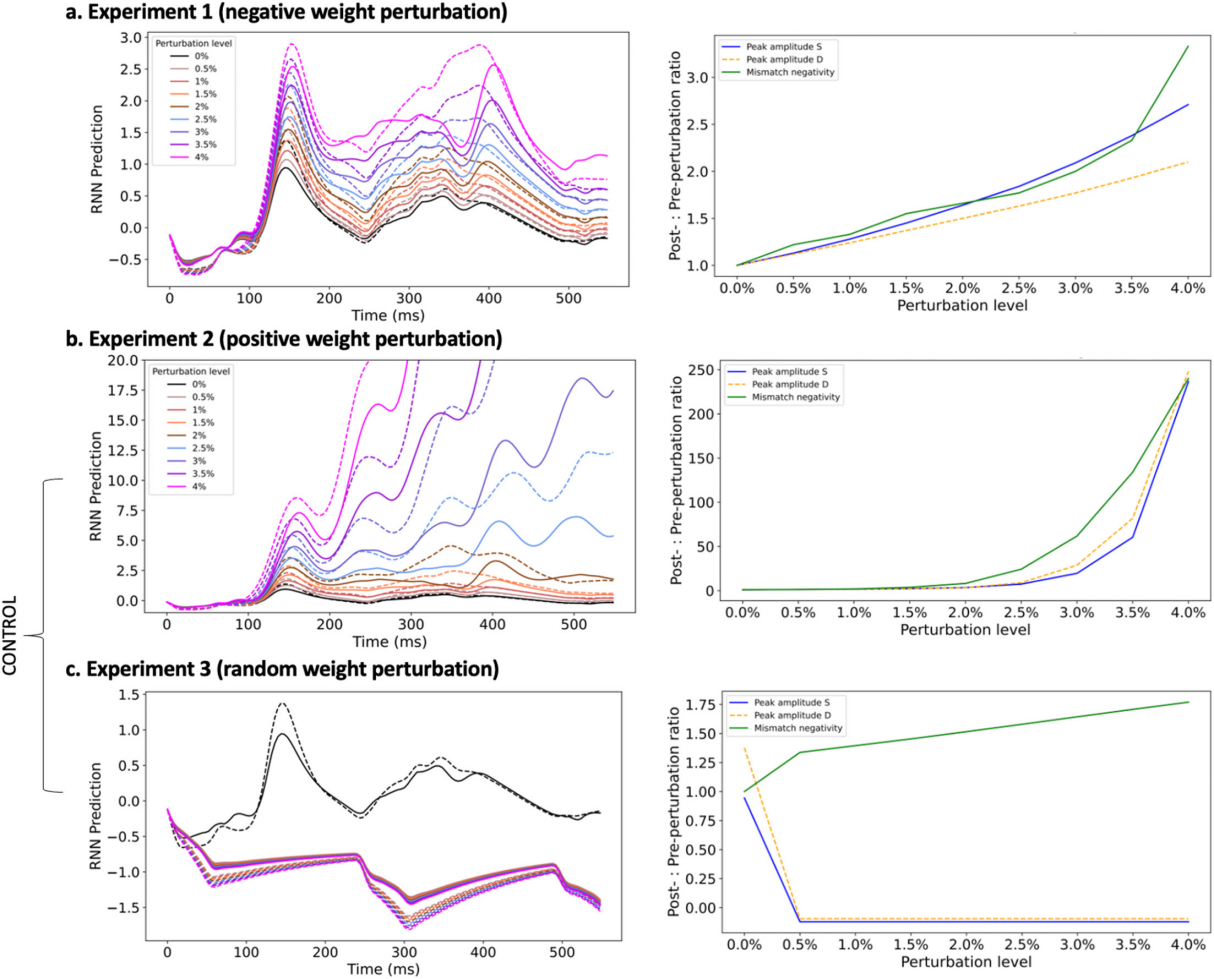
RNN predictions before and after each perturbation experiment. Predicted AEFs for standard and deviant inputs (left panel) and relative increases from initial RNN predictions (pre‐perturbation) after each perturbation experiment (right panel). (a) Experiment 1 (negative weight perturbation). (b) Experiment 2 (positive weight perturbation). (c) Experiment 3 (random weight perturbation).

To quantify how well the model fit to the data, we quantified the value of the loss function, which usually decreases with improved data fitting (i.e. smaller differences between network predictions and labels). The loss values evaluated on the test set, which implied both S and D inputs, are shown in Table 1. Experiment 1 (‘negative weight perturbation’) resulted in stable changes in the RNN output from the baseline, as highlighted by the lowest MSE values up to 3.5% inhibition reduction, with small MSE increases to the next perturbation level (Table 1, Figure 5a). Increasing excitation levels in experiment 2 (‘positive weight perturbation’) led to unstable RNN predictions reflecting runaway excitation (Table 1, Figure 5b). Experiment 3 (‘random weight perturbation’) resulted in relatively stable MSE values across the perturbation levels (Table 1, Figure 5c). These results showed that inhibition reduction experiments best fitted the evoked response data, with small increases in perturbation strength resulting in small increases in loss values, consistent with our predictions.

**TABLE 1:** RNN mean squared error (MSE) values during test set evaluation.

	Test loss function (MSE values)							
	Pre‐perturbation: 0.4							
	Perturbation level							
	0.5%	1%	1.5%	2%	2.5%	3%	3.5%	4%
Experiment 1	0.39	0.43	0.5	0.63	0.87	1.28	1.93	3.21
Experiment 2	0.44	0.71	1.72	6.31	53.23	368.7	2279.2	14323.8
Experiment 3	2.19	2.22	2.26	2.3	2.34	2.34	2.42	2.46

We next analysed the model's qualitative dynamical outputs under perturbation for the three experiments. The output unit predictions for S and D inputs were computed in each experiment and compared to baseline levels, pre‐perturbation (Figure 5). Inhibition reduction (experiment 1) resulted in predicted AEFs with linearly increasing amplitudes (Figure 5, Table 2) relative to the baseline levels, which mirrors the trend observed empirically between the control and ZDHHC9 groups (Figure 2). MMN showed nonlinear increases, especially at the highest perturbation levels. AEF peak latencies also increased from baseline, by 4 ms, and remained constant across the 0.5–2.5% perturbations before a further 4 ms increase at the 3–4% perturbations (Table 2). No differences in peak latencies were obtained between S trial predictions and D trial predictions (Table 2). Increased excitation (experiment 2) resulted in exponential increases in AEF peak amplitude from the 4th perturbation level onwards (Figure 5), accompanied by peak latencies in the latter half of the AEF window (Table 2). Perturbing a random set of weights (experiment 3) resulted in opposite polarity AEFs with peak amplitudes and MMN varying minimally across the perturbation levels and a constant peak latency at 300 ms (Figure 5, Table 2).

**TABLE 2:** AEF metrics before and after each level of perturbation for the three experiments.

a. Pre‐perturbation AEFs		
Trial type	Peak amplitude	Peak latency
S	0.94	140
D	1.37	140

b. Post‐perturbation AEFs										
			Perturbation level							
**Experiment**		**Trial type**	**0.5%**	**1%**	**1.5%**	**2%**	**2.5%**	**3%**	**3.5%**	**4%**
**1**	Peak amplitude	S	1.07	1.21	1.37	1.55	1.74	1.98	2.25	2.56
		D	1.54	1.71	1.88	2.07	2.25	2.43	2.65	2.89
	Peak latency	S	144	144	144	144	144	148	148	148
		D	144	144	144	144	144	148	148	148
	MMN		0.11	0.12	0.14	0.15	0.16	0.17	0.2	0.3
**2**	Peak amplitude	S	1.26	1.65	2.12	3.3	6.97	18.48	57.1	223.4
		D	1.81	2.3	2.8	4.5	12.4	39.65	112.7	341.8
	Peak latency	S	144	144	148	400	500	508	544	544
		D	144	144	144	344	516	544	544	544
	MMN		0.13	0.18	0.35	0.77	2.3	5.87	12.7	22.8
**3**	Peak amplitude	S	−1.38	−1.39	−1.41	−1.42	−1.43	−1.44	−1.46	−1.48
		D	−1.61	−1.64	−1.67	−1.69	−1.72	−1.75	−1.78	−1.8
	Peak latency	S	300	300	300	300	300	300	300	300
		D	300	300	300	300	300	300	300	300
	MMN		0.12	0.13	0.13	0.14	0.15	0.15	0.16	0.17

These outcomes can be traced to the ReLU activation function for the RNN units, which loosely mimics the all‐or‐nothing property of biological neurons. In experiment 1, the network output changes gradually as the inhibitory weights are reduced and more neurons cross the activation threshold. In experiment 2, when excitatory weights are amplified, large outputs propagate through the multi‐layered network, resulting in runaway network activity. In experiment 3, the decrease in inhibition balances the increase in excitation and thus the RNN predictions remain stable across perturbation levels.

### 3.4 Validating the Effect of Weight Perturbation Experiments on RNN Latent Dynamics

Principal component analysis was performed on the post‐perturbation activations of the 4th hidden layer after model evaluation on the test set (Figure S4). This provided a visualisation of the internal representation of the RNN just before the outputs are read by the output unit and are in line with the results in Table 2. Experiment 1 resulted in stable predictions across the perturbation levels, as activation in latent space at the first prediction timepoint was in proximity of activation level at the final prediction timepoint, as shown in the latent activity depicted in Figure S4a. The area covered by the latent space activity increased with the perturbation levels, reflecting the AEF amplitude increases (Figure S4a). The latent activity in experiment 2, starting from the 5th perturbation level (2.5%) onwards resulted in values that maximally differed between the initial and final timepoints, highlighting runaway activity (Figure S4b). Experiment 3 resulted in different internal representations which remained stable across the perturbations (Figure S4c).

## 4 Discussion

This study tested the hypothesis that empirical neurophysiological differences between a monogenic neurodevelopmental disorder group (*ZDHHC9*‐associated ID) and control group are compatible with reduced inhibition in a network model of auditory processing. We observed that reduction in inhibition levels within an RNN model resulted in increasing peak amplitudes and peak latencies of model outputs, which qualitatively matched the case–control results. In contrast, increasing excitation or perturbing a random set of connections resulted in a phase shift of the RNN output, inconsistent with empirically derived results.

Empirical MEG analyses focused on mMMN to investigate whether *ZDHHC9* variants are associated with differences in adaptive auditory processing. Two prominent theories have been proposed to explain the mMMN. One view of the auditory MMN, Näätänen's echoic memory theory, posits that it links sensory processing to higher‐level cognitive functions by detecting violations in stimulus sequences, which are compared to information stored in ultra‐short‐term (echoic) memory (Garrido et al. 2009; Näätänen 1990; Näätänen and Winkler 1999; Näätänen 2000). Another view, aligned with the Bayesian Brain Hypothesis, suggests that the MMN represents a prediction error signal (Holmes and Nolte 2019; Fitzgerald and Todd 2020), reflecting the brain's continuous process of learning environmental statistics, detecting regularities and changes, and generating top‐down predictions to compare against incoming stimuli (Garrido et al. 2009; Friston 2005). In the current study, the ZDHHC9 group showed deviant‐related activity in the right temporal cluster that was more spatially extensive and of greater amplitude compared to controls. These findings could be interpreted in light of both theories. From the perspective of Näätänen's model, the prolonged and enhanced responses may reflect differences in echoic memory maintenance or in the fidelity of comparing deviant stimuli to recent sensory traces. Alternatively, the findings could align with the Bayesian Brain Hypothesis, suggesting that *ZDHHC9* variants may lead to altered prediction error signalling, with heightened responses reflecting changes in the brain's ability to detect and adapt to environmental regularities. Previous source modelling of mMMN has identified bilateral sources in the superior temporal gyrus and inferior frontal gyrus, suggesting that mMMN generation relies on bidirectional frontotemporal connectivity (Garrido et al. 2008; Port et al. 2016). These regions, which are implicated in both echoic memory and predictive coding, likely underlie the observed group differences. While the exact mechanisms remain uncertain, our findings highlight the need for future studies to further disentangle the contributions of echoic memory and predictive coding to mMMN alterations in individuals with *ZDHHC9* variants. The current observations remain qualitative as direct, between‐group comparison was challenging due to limited number of channels for which both groups demonstrated a significant mMMN. Future studies using the roving oddball paradigm may be additionally informative if level of attention and distraction are controlled, via an active response paradigm in addition to passive stimulation during silent movie watching (supplemented by post‐movie questions). It may also be relevant that we chose to baseline correct data for a relatively long pre‐stimulus period, to confirm no systematic differences in pre‐stimulus activity for S and D events. It will be important to replicate these results in larger samples of individuals with *ZDHHC9* variants, within specific age‐bands, and in comparison to additional groups such as other monogenic causes of ID. However, a limitation of rare conditions research is that sample sizes are inevitably small—the current study involved all individuals known to have *ZDHHC9*‐associated ID living in the UK at the time of data collection (except for one individual with severe ID).

To explore the network origins of observed between‐group differences in MEG signal generation, we applied RNN modelling, in which the activity of hidden units resembles that of neural populations (O'Reilly 2022; O'Reilly, Angsuwatanakul, and Wehrman 2022). During model training, the RNN weights representing connections between the model units were optimised so that the RNN predictions most closely matched the labels (the grand‐average AEF waveforms generated by adding noise to the control group AEF). The feedforward and recurrent weight matrices obtained after training could be interpreted as analogous to wiring patterns supporting neurotypical AEF generation. The RNN model enabled the comparison between its predictions and neurophysiological responses given several analogies that could be made between the two. For instance, creating an RNN model with multiple hidden layers enabled the signal propagation through a hierarchical structure comprising of feedforward and feedback interactions, similar to sensory processing. As the information propagated through the layers, the pattern of activations became more complex, similar to neural signals travelling from sensory periphery to subcortical structures and regions of the sensory cortex. Moreover, the RNN approach facilitated the formation of a high‐dimensional activation space given the total of 256 hidden units, which attempted to mimic signals arising from a large number of underlying neural sources that underlie scalp‐recorded AEFs.

The RNN model was used as a platform to test a mechanistic hypothesis underpinning AEF generation in the ZDHHC9 group, specifically reduced inhibition arising from enzyme ZDHHC9 dysfunction. Inhibitory weight perturbation of the model after training resulted in increased AEF and deviant‐related responses, in keeping with empirical observations although with some limitations. While the model perturbations resulted in an increased synaptic E:I ratio that is smaller than observations in primary cultures with *ZDHHC9* knockout (Shimell et al. 2019), they were appropriate for the current RNN model which captures features of sensory processing, whilst omitting biological details such as separate excitatory and inhibitory units and features of connectivity. Another limitation of the model is that it did not consider developmental effects. Genetic effects usually interact with the environment continuously, and this interaction shapes sensory processing and behaviours. A future modelling approach would take this aspect into account and might include perturbations from the start of model training as opposed to post‐training, potentially in the form of a regularisation term within the loss function equation or a time‐varying learning rate hyperparameter.

The results of the current study go a step further toward understanding the associations between *ZDHHC9* loss of function, seizure susceptibility and developmental language difficulties. A role for hyperexcitability and atypical AEFs in language development has been proposed in the context of autism, where M100 latencies are persistently delayed and predict language improvement over time (Port et al. 2016). M100 latency has been proposed as a marker for capacity to improve cognitive skill, and language skill in particular, relating to cortical interneuron development and GABA concentration (Port et al. 2017). Of note, rare variants in GABA receptor subunits have been associated with risk for rolandic epilepsy, potentially bridging seizure risk and cortical inhibition relevant to cognitive development (Reinthaler et al. 2015). However, it is not known whether M100 amplitude, besides latency, reflects properties of the auditory cortex involved in language acquisition. This could be explored in future prospective studies across monogenic causes of RE, incorporating behavioural measures of speech and auditory processing for correlation with MEG (Smith et al. 2017). The existing literature provides discrepant accounts of how MMN is affected in epilepsy patients and those affected by developmental language disorders. Some studies revealed links between developmental language disorders and diminished MMN amplitudes, which points to a causal relationship between inefficient auditory processing, insensitivity to phonetic cues and impaired speech and language skills (Baldeweg et al. 1999; Datta et al. 2010; Ahmmed, Clarke, and Adams 2008; Shafer et al. 2005). Furthermore, lower MMN amplitude has also been observed in children with rolandic epilepsy, with or without language impairments (Boatman et al. 2008; Metz‐Lutz and Filippini 2006; Filippini et al. 2015). In contrast, several studies have shown larger MMN responses in epileptic patients in response to pure tones (Miyajima et al. 2011; Gene‐Cos et al. 2005; Usui et al. 2008). Higher MMN amplitudes in people with epilepsy could indicate increased activation of the same neuronal population as in controls or activation of additional neuronal resources (Myatchin et al. 2009). Similar trends have been observed in children with learning difficulties and dyslexia compared to controls (Bishop 2007). These contrasting previous results may reflect small sample sizes and reproducibility issues or could reflect real differences in cortical processing contributing to language difficulties dependent on aetiology and associated network disturbances, which could be explored in future studies.

## 5 Conclusions

In summary, this study characterises neurophysiological processing in the *ZDHHC9*‐associated neurodevelopmental condition and provides a proof‐of‐concept for using neural network models to investigate the mechanistic origins of cognitive disorders. Our findings suggest that reduced inhibition is a plausible mechanism underlying the neurophysiological deviations observed in the *ZDHHC9* loss‐of‐function compared to controls, including delayed AEF peak latency, increased AEF peak amplitude, and heightened MMN responses. While these findings remain qualitative due to the small sample size, this study aims to inspire future experimental and computational investigations into how synaptic excitation‐inhibition imbalances influence cognitive function.

Future studies will ideally increase the complexity of the neural network models to better mimic sensory processing and apply these in larger datasets of individuals with cognitive impairments arising from a range of genetic variations. It will also be important to perform studies at more granular scales, for example by studying rodent models of these monogenic disorders and obtaining single‐neuron recordings that would shed light on the neuronal dynamics in these conditions. A wider range of cognitive and memory tasks implemented with rodent models and parallel human studies would also be useful to explore effects of single gene variants on learning and memory, together with underlying alterations in neuronal activity and connectivity. Collectively, these inter‐disciplinary studies will contribute to improved multi‐level understanding of monogenic conditions impacting on cognition and will potentially inform future therapeutic interventions in relevant clinical populations.

## Author Contributions

RIV conceived the study, analysed data, and wrote the manuscript. JA and DA advised on analyses. DA and KB supervised the project. All authors reviewed, edited, and approved the manuscript for submission.

## Ethics Statement

Ethical approval for this study was granted by the Cambridge Central Research Ethics Committee (11/0330/EE: Phenotypes in Intellectual Disabilities). Written informed consent was provided by all participants, or a parent/carer for participants under 16 years of age.

## Conflicts of Interest

The authors declare no conflicts of interest.

### Peer Review

The peer review history for this article is available at https://www.webofscience.com/api/gateway/wos/peer‐review/10.1111/ejn.70124.

## Supporting information


**Figure S1** Auditory‐evoked neuromagnetic fields (AEF) across all stimuli and corresponding topographic maps.
Figure S2 Frequency‐latency dependence of auditory‐evoked neuromagnetic fields (AEF).
Figure S3 Direct comparison of mismatch responses in ZDHHC9 and control groups.
Figure S4 Latent dynamics of 4th hidden layer.

## Data Availability

Data are available on request due to privacy/ethical restrictions.
