# Supplementary material for: Synaptic Function and Sensory Processing in ZDHHC9‐Associated Neurodevelopmental Disorder: A Mechanistic Account
Source: Eur J Neurosci. 2025 May 1;61(9):e70124. doi: 10.1111/ejn.70124 (PMC12044517; doi:10.1111/ejn.70124)
Supplement: Supplementary file 1 — Figure S1 Auditory‐evoked neuromagnetic fields (AEF) across all stimuli and corresponding topographic maps. Figure S2 Frequency‐latency dependence of auditory‐evoked neuromagnetic fields (AEF). Figure S3 Direct comparison of mismatch responses in ZDHHC9 and control groups. Figure S4 Latent dynamics of 4th hidden layer. [file EJN-61-0-s001.docx]

**Figure S1: Auditory evoked neuromagnetic fields (AEF) across all stimuli and corresponding topographic maps**


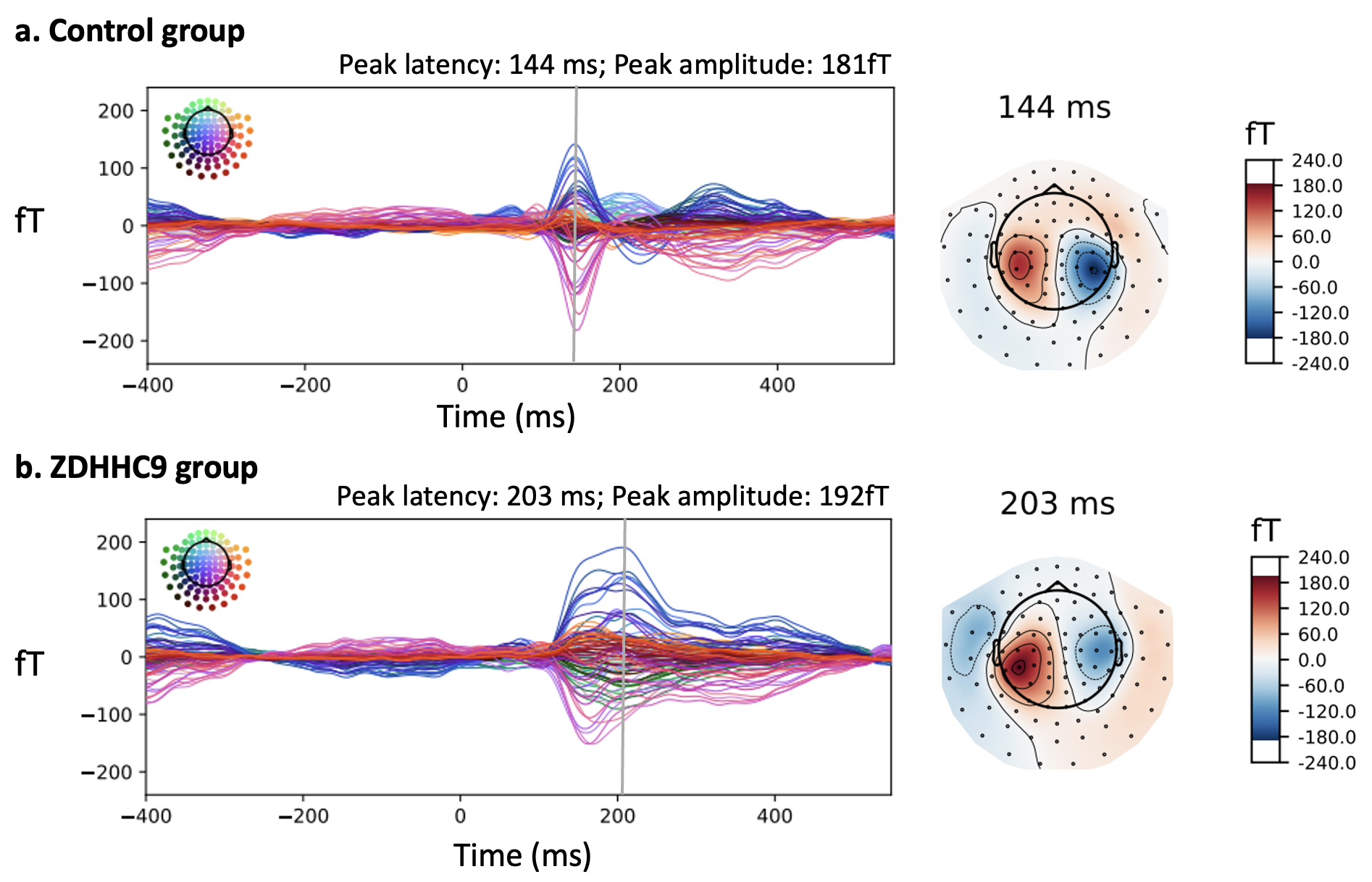


**Figure S2: Frequency-latency dependence of auditory evoked neuromagnetic fields (AEF)**

**
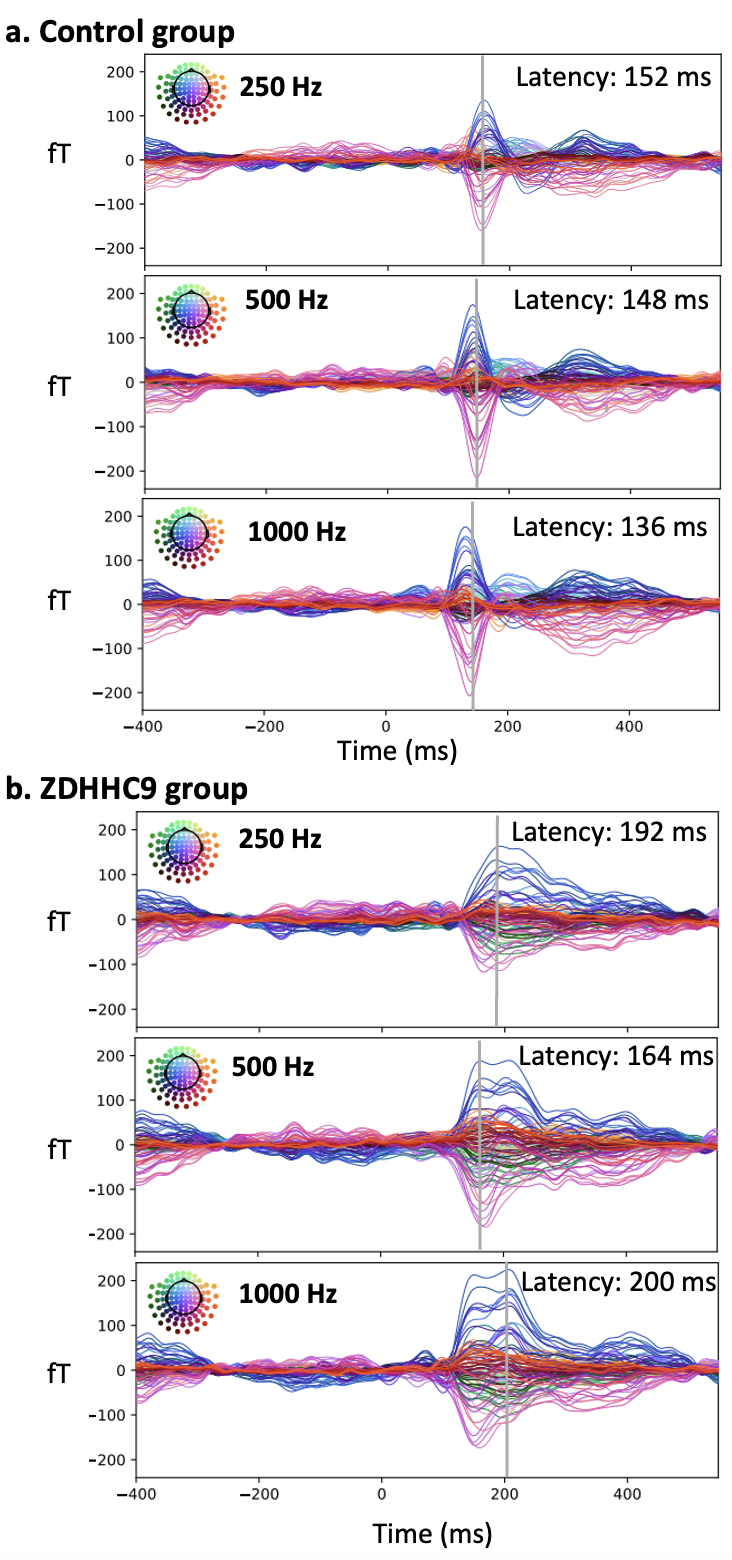
**

**Figure S3: Direct comparison of mismatch responses in ZDHHC9 and control groups**

**
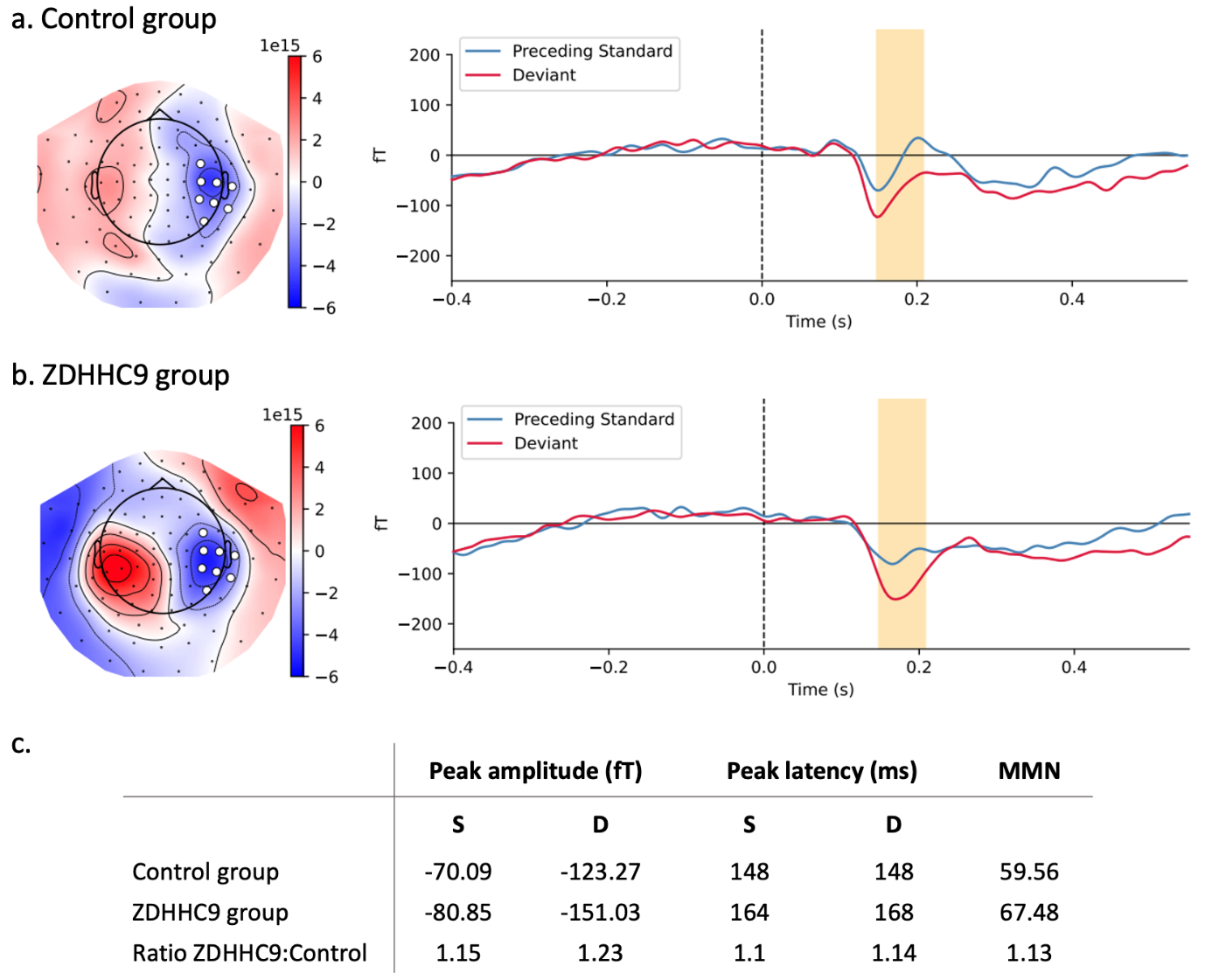
**

Evoked responses to all deviants (D) and their corresponding preceding standard (S) at the eight sensors where significant S-D differences (in yellow) were found in both the control and ZDHHC9 groups. In yellow, the timeframe where the S-D differences are significant in both groups is shown. **a.** Control group response at the eight overlapping channels of the significant cluster (*p*-value = 0.0008). **b.** ZDHHC9 group response at the same eight channels of the significant cluster in this group (*p*-value = 0.0015). **c.** The values from the plots in a. and b. (absolute values for peak amplitudes) and mismatch negativity calculated as mean absolute error between standard-evoked responses and deviant-evoked responses in the significant time window.

**Figure S4: Latent dynamics of 4^th^ hidden layer**


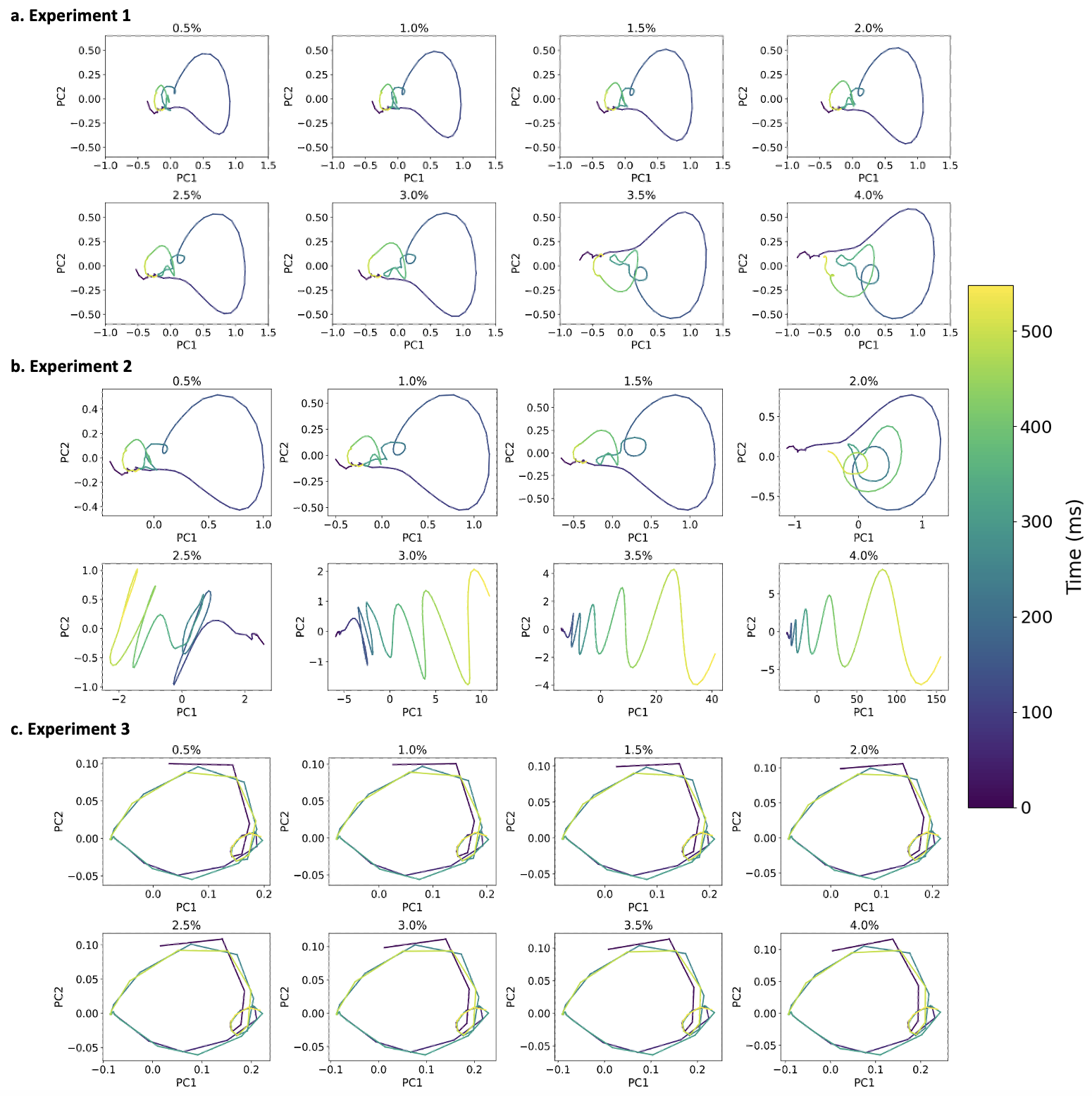


Principal component analysis was performed on the activations of the last hidden layer, which resulted in a latent activity trajectory over time.
